# LncRNA MIAT impairs cardiac contractile function by acting on mitochondrial translocator protein TSPO in a mouse model of myocardial infarction

**DOI:** 10.1038/s41392-021-00538-y

**Published:** 2021-05-03

**Authors:** Xue Bai, Chao Yang, Lei Jiao, Hongtao Diao, Ziyu Meng, Lei Wang, Hao Cui, Lihua Sun, Yong Zhang, Baofeng Yang

**Affiliations:** 1 Department of Pharmacology (State-Province Key Laboratories of Biomedicine-Pharmaceutics of China and Key Laboratory of Cardiovascular Medicine Research, Ministry of Education), College of Pharmacy, Harbin Medical University, Harbin, Heilongjiang P. R. China; 2 Institute of Metabolic Disease, Heilongjiang Academy of Medical Science, Harbin, China

**Keywords:** Non-coding RNAs, Cardiology

**Dear Editor**,

Long non-coding RNA MIAT (lncR-MIAT) has recently been identified as a risk factor for myocardial infarction (MI).^1^ However, how lncR-MIAT controls MI remained yet to be determined. To shed light on this issue, we firstly detected the expression of lncR-MIAT using qRT-PCR and found a robust elevation (>5-fold) of lncR-MIAT level in heart of MI mice relative to sham-operated control counterparts (Supplementary Fig. S1a). This upregulation of lncR-MIAT was diminished after injection of lentivirus carrying a shRNA of lncR-MIAT (Lv-shMIAT) to silence endogenous lncR-MIAT in myocardium. Similarly, 3-fold increase of lncR-MIAT was observed in cultured neonatal mouse ventricular cells (NMVCs) in response to hypoxic insult, which was effectively mitigated by siRNA (siMIAT; Supplementary Fig. S1b) that effectively silenced endogenous lncR-MIAT in NMVCs (Supplementary Fig. S1c).

Echocardiographic measurements in MI mice demonstrated that knockdown of lncR-MIAT by Lv-shMIAT significantly restored the impaired cardiac function, as manifested by the reinstatement toward normal values of ejection fraction (EF) and fractional shortening (FS) (Supplementary Fig. S1d–h and Supplementary Table 1).

To understand how knockdown of lncR-MIAT improves cardiac function, we measured the number of TUNEL-positive cells to quantify apoptotic cell death and found that it was robustly increased in MI mice compared to that in the sham group, and notably this increase was essentially prevented by pretreatment with Lv-shMIAT (Supplementary Fig. S2a).

We then employed Western blot analysis to measure the alterations of the total protein levels of caspase3 (Casp3) and cleaved or activated caspase3 (c-Casp3), the executioner of apoptosis. Our results demonstrated that while Casp3 was enormously upregulated in both of its expression and activation in MI hearts, the increases were markedly mitigated by Lv-shMIAT (Supplementary Fig. S2b, c).

To gain further insight into the mechanisms, we conducted electron microscopic (EM) assessments. The results unambiguously exhibited that MI caused pronounced mitochondrial membrane damages in the peri-infarct zone of myocardium with mitochondria swelling and disrupted mitochondrial surface, whereas knockdown of lncR-MIAT rescued the damages (Supplementary Fig. S3a). Opening of mitochondrial permeability transition pore (mPTP) can depolarize mitochondrial membrane potential (ΔΨm) to trigger cytochrome c (Cyt-C) release into cytosol, eventually leading to apoptosis.^2^ Translocator protein (TSPO), a mitochondrial membrane protein in cardiac cells, plays a crucial role in controlling mitochondrial membrane potential by inducing opening of mPTP to trigger the subsequent signaling events: TSPO↑ → mPTP opening↑ → ΔΨm↓ → Cyt-C release↑ → c-Casp3↑ → Apoptosis↑.^3,4^ All these findings prompted us to perform the following experiments.

First, TSPO mRNA and protein levels were found significantly increased in MI, which was nearly abolished upon silencing of lncR-MIAT (Supplementary Fig. S3b and Fig. 1a, b). Calcein/CoCl_2_-quenching assay showed that in NMVCs cultured under hypoxic conditions, calcein signal (green fluorescence) disappeared from its normal localization within mitochondria seen under normoxic conditions, which was restored by MIAT siRNA (siMIAT) (Fig. 1c), indicating that silence of lncR-MIAT prevents mPTP opening thereby protecting mitochondrial membrane integrity from hypoxic damages. Moreover, application of TPSO activator FGIN-1-27 countered the inhibitory effect of siMIAT on mPTP opening.

**Fig. 1:**
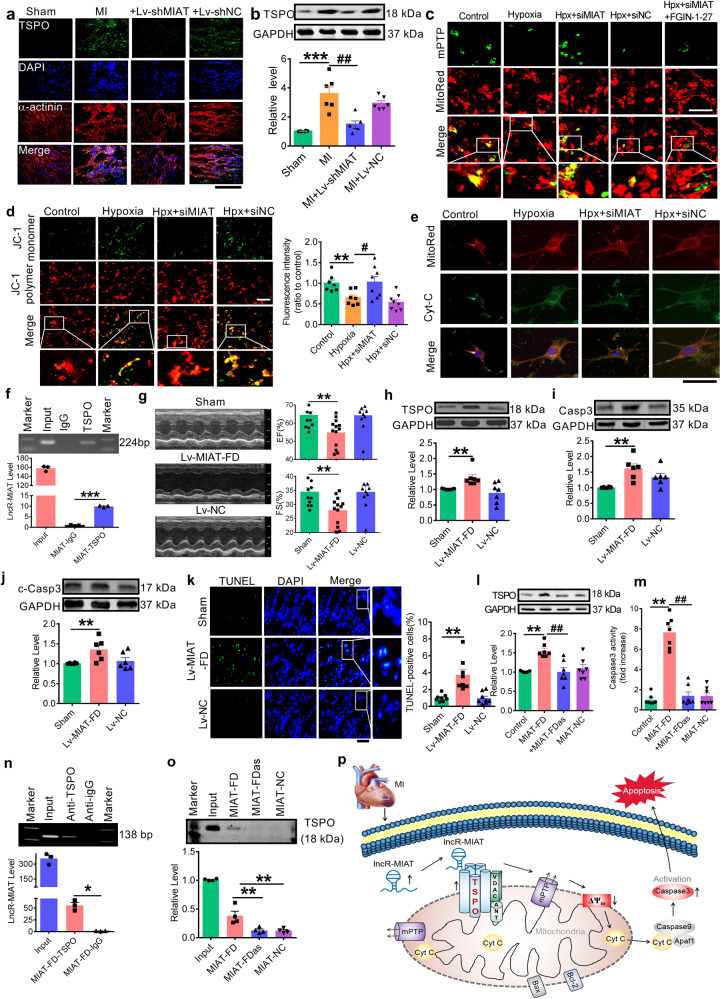
**a** Representative images of immunofluorescence staining of mitochondrial translocator protein (TSPO in green) expression in heart tissue sections of MI mice (600× magnification). DAPI staining was used for identifying nucleus and α-actinin staining for cardiac muscles. Note that MI induced substantial expression of TSPO, which was suppressed by lentivirus vector carrying a short RNA sequence targeting long non-coding RNA lncR-MIAT (Lv-shMIAT) to silence endogenous expression of lncR-MIAT in vivo with Lv-shNC as a negative control. **b** Upregulation of TSPO protein level in MI hearts and restoration of this upregulation by Lv-shMIAT, but not by Lv-shNC, as assessed by Western blot analysis. ***P < 0.001 vs. Sham; ^##^*P* < 0.01 vs. MI; *n* = 5–6. **c** Typical examples of calcein-AM staining (200× magnification) showing the changes of opening of mitochondrial permeability transition pore (mPTP) in cultured neonatal mouse ventricular cells (NMVCs). mPTP was stained in green, and the loss of staining indicates the opening of mPTP. Note that hypoxia triggered opening of mPTP, and this change was reversed by lncR-MIAT siRNA (siMIAT) to knockdown endogenous lncR-MIAT in vitro, but not by siNC as a negative control construct for siMIAT, which was blocked by the addition of TSPO activator FGIN-1-27. MitoRed staining was used to localize mitochondria. **d** Left panel: representative images (200× magnification) of JC-1 staining depicting the changes of the mitochondrial membrane potential in NMVCs. The green staining of JC-1 monomer seen under hypoxic conditions indicates depolarization of mitochondrial membrane potential, and the red staining of JC-1 polymer marks mitochondria. Right panel: mean ± SEM of the intensity of JC-1 monomer staining showing the restoration of the hypoxia-induced depolarization of mitochondrial membrane potential by siMIAT. **P < 0.01 vs. Control; ^#^*P* < 0.05 vs. Hypoxia; *n* = 7–8. **e** Representative images of immunofluorescent staining (600× magnification) for cytochrome c (Cyt-C) in NMVCs. Cyt-C (green) was restricted within the rod-shaped bodies stained in red by MitoRed, indicating its colocalization to mitochondria under normoxic conditions. Under hypoxia, the distribution of Cyt-C became diffused throughout the cytoplasm indicting its release from mitochondria, which was effectively prevented by siMIAT. **f** A representative image of RIP assay showing the immunoprecipitation of lncR-MIAT by TSPO antibody. The bottom panel provides the statistical data (mean ± SEM) on the level of lncR-MIAT immunoprecipitated with TSPO antibody determined by qPCR. IgG was used as a negative control, and the data were normalized to IgG. Input was used as a positive control. ***P < 0.001 anti-TSPO vs. anti-IgG; *n* = 3. **g** Overexpression of a 226-nt fragment encompassing the highly conserved sequence region of lncR-MIAT (MIAT-FD for functional domain of lncR-MIAT) by infection of lentivirus carrying MIAT-FD (Lv-MIAT-FD) in healthy mice caused cardiac dysfunction, as reflected by the decreased EF% and FS%. **P < 0.01 vs. Sham; *n* = 10–13. **h** Western blot analysis showing the increased TSPO expression at the protein level with overexpression of MIAT-FD elicited by Lv-MIAT-FD, but not by its negative control construct Lv-NC (scrambled negative control). **P < 0.01 vs. Sham; *n* = 7. **i** Overexpression of MIAT-FD upregulated the protein level of total caspase3 (Casp3), relative to sham-operated control and Lv-NC-treated control mice. **P < 0.01 vs. Sham; *n* = 6. **j** Overexpression of MIAT-FD upregulated the protein level of cleaved caspase3 (c-Casp3), relative to sham-operated control and Lv-NC-treated control mice. **P < 0.01 vs. Sham; *n* = 6. **k** Representative images of TUNEL staining of mouse myocardial sections for apoptotic cells (200× magnification). Note that overexpression of MIAT-FD in healthy mice infected with Lv-MIAT-FD, but not with Lv-NC, robustly increased the TUNEL-positive cells. Apoptotic cells were semi-quantified and expressed as the percentage of TUNEL-positive cells over DAPI stained cells. **P < 0.01 vs. Sham; *n* = 8. **l** Western blot results showing the significant elevation of TSPO protein level in NMVCs transfected with MIAT-FD and the reversal of TSPO deregulation by co-transfection of the antisense oligo fragment of MIAT-FD (MIAT-FDas). MIAT-NC: the negative control for MIAT-FD. **P < 0.01 vs. Control; ^##^*P* < 0.01 vs. MIAT-FD; *n* = 7. **m** Forced expression of MIAT-FD robustly increased caspase3 activity in NMCMs as determined by caspase3 assay kit. This detrimental effect of MIAT-FD was abrogated by MIAT-FDas. MIAT-NC showed no effects on caspase3 activity. **P < 0.01 vs. Control; ^##^*P* < 0.01 vs. MIAT-FD; *n* = 7. **n** RNA immunoprecipitation (RIP) analysis showing the precipitation of MIAT-FD by TSPO antibody with IgG as a negative control. Input: positive control. *P < 0.05 anti-TSPO vs. a**n**ti-IgG; *n* = 3. **o** RNA pulldown analysis showing the pulldown of TSPO protein by MIAT-FD, but not by MIAT-FDas or MIAT-NC. Input: positive control. **P < 0.01 vs. MIAT-FD; *n* = 4. **p** Schematic diagram depicting the proposed signaling mechanisms of lncR-MIAT in the setting of MI: MI → lncR-MIAT ↑ → TSPO ↑ → mPTP opening ↑ → ΔΨm ↓ → Cyt-C release ↑ → c-Casp3 ↑ → apoptosis ↑

We next verified the ability of siMIAT to alleviate hypoxia-induced decline of ΔΨm using JC-1 staining. As illustrated in Fig. 1d, siMIAT prevented the loss of ΔΨm with significant increase in the ratio of red/green fluorescence intensity.

Release of Cyt-C from mitochondria into cytoplasm is a key indicator for the activation of mitochondrial death pathway due to the loss of mitochondrial membrane potential.^5^ As depicted in Fig. 1e, siMIAT attenuated the cytoplasmic distribution of Cyt-C (decrease in green staining) and maintained its mitochondrial localization (increased yellow staining within mitochondria) in hypoxic cardiomyocytes, indicating that knockdown of lncR-MIAT suppressed Cyt-C released from mitochondria.

To decipher the molecular mechanisms by which lncR-MIAT regulates TSPO, we conducted bioinformatics prediction using the database—RPISeq (http://pridb.gdcb.iastate.edu/RPISeq/#) and identified a potential of direct interaction between lncR-MIAT and TSPO protein (Supplementary Fig. S4). Subsequent RIP assay revealed significant enrichment of lncR-MIAT in the anti-TSPO complex fraction relative to the anti-IgG fraction (Fig. 1f). Fish staining of lncR-MIAT and TSPO in NMVCs yielded further evidence for the in situ interaction between lncR-MIAT and TSPO:lncR-MIAT and TSPO were co-localized in the mitochondria (Supplementary Fig. S5).

Given that lncR-MIAT could directly interact with TSPO by RNA:protein binding, we reasoned that a nucleotide fragment encompassing the region of lncR-MIAT that binds TSPO should reproduce the effects of the full-length lncR-MIAT on TSPO, mitochondria, apoptosis, and cardiac function. We examined this hypothesis by taking the following steps.

We first analyzed the TSPO-binding region of lncR-MIAT sequence and found a domain that falls right within it that is highly conserved among varying species. Intriguingly, this is the only one homologous region among the four human sapiens lncR-MIAT variants (Accession: NR_003491.3, NR_033319.2, NR_033320.2, and NR_033321.2) and one Mus musculus lncR-MIAT sequence (Accession: NR_033657.1; locus 6101–6326 of chromosome 22q13.31) (Supplementary Fig. S6a).

We then synthesized a 226-nt fragment of the conserved sequences containing the predicted TSPO-binding region (for convenience, we named this fragment the functional domain of lncR-MIAT or MIAT-FD for abbreviation) and engineered MIAT-FD into lentivirus vector (Lv-MIAT-FD). Lv-MIAT-FD or Lv-NC (negative control) was directly injected into the mouse left ventricular cavity, and 7 days later, overexpression of MIAT-FD (an approximate 6-fold increase relative to mock-treatment) was verified (Supplementary Fig. S6b). As anticipated, overexpression of Lv-MIAT-FD reproduced MI-like detrimental phenotypes in healthy mice with significant decreases in EF% and FS%, compared to mock-treated or Lv-NC control mice (Fig. 1g). Resembling the alterations seen in MI, Lv-MIAT-FD caused robust increases in TSPO mRNA and protein levels in healthy mice treated relative to mock-treated or Lv-NC (Supplementary Fig. S6c and Fig. 1h). Furthermore, Lv-MIAT-FD prominently increased the levels of total Casp3 and c-Casp3, strongly indicating an induction of apoptosis (Fig. 1i, j). Consistently, Lv-MIAT-FD induced substantial cardiomyocyte apoptosis in healthy mice (Fig. 1k).

Similar alterations were reproduced in NMVCs. First, transfection of the plasmid for MIAT-FD overexpression remarkably increased TSPO protein level, but the negative control construct produced no effect (Fig. 1l). Second, MIAT-FD significantly increased Casp3 activity (Fig. 1m). Finally, MIAT-FD robustly increased apoptotic cells (Supplementary Fig. S7). In all cases, co-transfection of antisense fragment to MIAT-FD (MIAT-FDas) abrogated the deleterious effects of MIAT-FD (Fig. 1l, m and Supplementary Fig. S7).

We then confirmed the direct MIAT-FD:TSPO association similar to the lncR-MIAT:TSPO binding using RIP assay. As depicted in Fig. 1n, there was a significant enrichment of MIAT-FD in anti-TSPO complex fraction. Moreover, pulldown of MIAT-FD was accompanied by a significant quantity of TPSO, which was tremendously abrogated by MIAT-FDas (Fig. 1o).

In conclusion, our results revealed that MIAT is a pro-apoptotic lncRNA by targeting TSPO to damage mitochondria and triggering the mitochondrial death pathway (Fig. 1p), and MIAT-FD is likely the functional motif responsible for the deleterious action of the full-length lncR-MIAT. LncR-MIAT interference may therefore be considered a potential new approach for the treatment of MI. The limitation of our study was that we did not explain clearly how lncR-MIAT was upregulated after MI. Possible explanations for this are that hypoxia/ischemia induced the changes of the transcription factors of lncR-MIAT or caused the alteration of epigenetic modification of lncR-MIAT, which consequently affect the expression of lncR-MIAT. Yet, rigorous future studies are required to clarify these issues.

## Supplementary information

Supplementary material

## Data Availability

The data sets used and/or analyzed during the current study are available from the corresponding author on reasonable request.
