# Supplementary material for: LncRNA MIAT impairs cardiac contractile function by acting on mitochondrial translocator protein TSPO in a mouse model of myocardial infarction
Source: Signal Transduct Target Ther. 2021 May 3;6:172. doi: 10.1038/s41392-021-00538-y (PMC8093248; doi:10.1038/s41392-021-00538-y)
Supplement: Supplementary file 1 — Supplementary material [file 41392_2021_538_MOESM1_ESM.docx]

**Supplementary Materials for**

**LncRNA MIAT impairs cardiac contractile function by acting on mitochondrial translocator protein TSPO in a mouse model of myocardial infarction**

Xue Bai^1,†^, Chao Yang^1,†^, Lei Jiao^1,†^, Hongtao Diao^1^, Ziyu Meng^1^, Lei Wang^1^, Hao Cui^1^, Lihua Sun^1^, Yong Zhang^1,2,^*, Baofeng Yang^1,^*

^1^Department of Pharmacology (State-Province Key Laboratories of Biomedicine-Pharmaceutics of China, Key Laboratory of Cardiovascular Medicine Research, Ministry of Education), College of Pharmacy, Harbin Medical University, Harbin, Heilongjiang 150081, P. R. China; ^2^Institute of Metabolic Disease, Heilongjiang Academy of Medical Science, Harbin 150086, China. ^2^Institute of Metabolic Disease, Heilongjiang Academy of Medical Science, Harbin 150086, China.

^†^Authors with equal contributions to this study.

*Corresponding authors: Yong Zhang, hmuzhangyong@hotmail.com, or Baofeng Yang, yangbf@ems.harbmu.edu.cn; Department of Pharmacology (the State-Province Key Laboratories of Biomedicine-Pharmaceutics of China), Harbin Medical University, 157 Baojian Road, Nangang District, Harbin, Heilongjiang, China 150081; Tel: +86 451 8667-1354; Fax: +86 451 8667-1354.

**This file includes:**

Materials and Methods

Figures S1 to S7

Tables S1 to S3

**METHODS AND MATERIALS**

***Animals***

C57BL/6J male mice ranging from 10 to 12 weeks in age and weighing between 25-30 g each for studying myocardial infarction and pregnant C57BL/6J mice for neonatal cardiomyocytes isolation were provided by the Experimental Animal Center of Harbin Medical University. Use of animals was approved by the Ethic Committees of Harbin Medical University and conformed to the Guide for the Care and Use of Laboratory Animals published by the US National Institutes of Health (NIH Publication No. 85-23, revised 1996).

***Mouse Model of Myocardial Infarction (MI)***

Mice were anesthetized with sodium pentobarbital (60 mg/kg, i.p.) and their chests were opened to expose the hearts. The left descending coronary artery (LAD) was ligated with a 7/0 nylon suture at 2 mm below the border between left atrium and ventricle to create myocardial infarction (MI). The sham-operated mice for control underwent the same experimental procedures as the MI group but without ligation of LAD. Standard lead II electrocardiograph (ECG) was recorded during and after 1 h of the process of surgery. MI was confirmed by significant S-T segment elevation with ECG measurements. The mice were anesthetized for hemodynamic measurements and sacrificed by cervical dislocation one week after MI. Randomization and blinding were adopted in animal experiments. Dead mice were excluded from data analysis.

***Echocardiographic Assessment of Cardiac Function***

Mice were anesthetized by intraperitoneal injection of sodium pentobarbital (65 mg/kg) (Sigma, St Louis, MO, USA). Left ventricular (LV) function was assessed with two-dimensional guided M-mode and transthoracic echocardiography with an ultrasound machine Vevo®2100 High-Resolution Imaging system (Visual Sonics, Toronto, ON, Canada) equipped with a 10.0-MHz phase-array transducer. The animals were placed in the supine or lateral position on a warming pad with their body temperature being maintained at 37°C. Two-dimensional end-diastolic and end-systolic long-axis views of LV were standardized with inclusion of apex, posterior papillary muscle, mitral valve, and aortic root. End-diastolic and end-systolic areas were obtained by hand-tracings of the LV endocardial contours, according to the American Society of Echocardiography leading edge method. LV systolic diameter (LVSd) and LV diastolic diameter (LVDd) were measured, and LV end-diastolic volume (LVEDv), LV end-systolic volume (LVESv), LV ejection fraction (EF), and fractional shortening (FS) were calculated from M-mode recording. Numeric images of the heart were obtained in both parasternal long-axis and short-axis views.

***Electron Microscopic Examination***

Heart tissues dissected from peri-infarct area or cultured cardiomyocytes were fixed with 2.5% glutaraldehyde at 4°C overnight, and then immersed in 2% osmium tetroxide. After dehydrated in a graded series of ethanol concentrations, sections were stained with uranyl acetate and lead citrate. The samples were examined and photographed under a JEOL 1200 electron microscope (JEOL Ltd, Tokyo, Japan).

***Isolation and Primary Culturing of Neonatal Mouse Ventricular Cells (NMVCs)***

Ventricular tissues from 3-day old neonatal mice were cut into l to 2 mm^3^ after the hearts had been rapidly removed and then dissociated in 0.25% trypsin at 37°C for 10 min. Heart tissues were trypsinized until the tissues disappeared, and cell suspensions were then collected by centrifugation at 1500 rpm for 145 sec. The collected cells were resuspended in DMEM (Hyclone, Logan, Utah, USA) supplemented with 10% fetal bovine serum (Gibco, Grand Island, NY, USA) and penicillin (100 U/ml)/streptomycin (100 U/ml) and cultured at 37°C in 5% CO_2_ + 95% air in a humidified incubator. After 90 min for fibroblast adherence, the cell suspension was plated into a 6-well plate at 3×10^5^ cells per well with DMEM containing arabinoside (10 nM) to suppress cardiac fibroblasts.

***Construction of*** ***Lentiviral Vector and Plasmid Vector carrying the Functional Domain of LncR-MIAT***

The recombinant plasmid vector containing MIAT-FD sequence was verified by PCR detection and then co-transfected with the lentiviral vector packaging plasmid into 293T cells. After 48-72 hours, the virus was collected. The lentiviral vector carrying the functional domain of LncR-MIAT was obtained after concentration and purification. The negative control construct Lv-NC was obtained with the same process. The above construction was completed by Shanghai Genechem Co.,Ltd.

***Synthesis of Antisense Oligo Fragment to Functional Domain of LncR-MIAT (******MIAT-FDas)***

Primers of MIAT-FDas sequence were designed and used to catch the target gene. The recombinant plasmid vector containing MIAT-FDas sequence was constructed. After PCR identification, positive clones were selected for sequencing comparison and analysis, and the aligned MIAT-FDas recombinant plasmid was obtained. The above construction process was completed by Shanghai Genechem Co.,Ltd.

***Lentivirus Mediated shRNA Transfection***

LncR-MIAT shRNA and a negative control shRNA were designed and purchased from Invitrogen (Shanghai, China). Their sequences are provided in **Table S2**. The constructs were inserted into lentivirus plasmid vector pcDNA 3.1(+/-) (Invitrogen, Shanghai, China). The plasmid was transfected into 293T cells by liposome reagent to generate a recombinant lentiviral vector through homologous recombination in which the transgene is incorporated into the viral genome. For in vitro experiments, lentivirus was added to NMVCs culture medium to yield a final multiplicity of infection (MOI) of 1. For in vivo studies, lentivirus (1×10^9^ TU/ml; 25 μl diluted in 75 μl saline) was injected into the left ventricular cavity through the tip of the heart using a needle syringe. LAD ligation was performed after arteries had been clamped for 10 sec.

***TUNEL Staining of Apoptotic Cells***

To determine the apoptosis levels in cardiomyocytes and heart tissues, we used the Terminal deoxynucleotidyl transferase dUTP nick end labeling (TUNEL) staining for detection. According to the instructions provided by TUNEL detection kit (Roche, Penzberg, Germany), the sectioned heart tissues (the peri-infarct areas of MI mice) or cells were fixed with 4% paraformaldehyde for 1 h, and then permeabilized with 0.1% Triton X-100 in 0.1% sodium citrate for 2 min. After washing with PBS three times, the sections were incubated with TUNEL reaction mixture at 37°C for 1 h, followed by staining of nuclei with DAPI. Five random fields for each single sample were analyzed by using laser scanning confocal microscopy (FV300; Olympus, Tokyo, Japan), and apoptotic cells were calculated as a percentage of the total number of nuclei.

***JC-1 Staining of Mitochondrial Membrane***

Mitochondria membrane potential (ΔΨm) was analyzed by using the 5,5’,6,6’-tetrachloro-1,1’,3,3’-tetraethyl-benzimidazolylcarbocyanine iodide (JC-1), an indicator which fluoresces red in cells with high ΔΨm while green in low-potential mitochondria. Briefly, cardiomyocytes were seeded in 24-well plates and incubated with JC-1 at 37°C for 20 min. After three washes, the cells were analyzed immediately with laser scanning confocal microscopy (FV300; Olympus, Tokyo, Japan) at an emission wavelength of 530 nm for green fluorescence and 590 nm for red one. Five random fields for each sample were selected for calculating the red/green ratio by using Image J software (NIH, Bethesda, MD, USA).

***Measurement of Cytochrome C Release***

Cardiomyocytes were incubated with 200 nM MitoTracker Red CMXROS (M-7512; Molecular Probes) to label mitochondria at 37°C for 30 min. After fixed with 4% paraformaldehyde for 15 min, permeabilized with 0.1% Triton X-100 for 20 min and further blocked with goat serum for 30 min, the cells were incubated with anti-cytochrome-C antibody at 4°C overnight. Next, the samples were incubated with Alexa Fluor 488-conjugated secondary antibody (Invitrogen, Carlsbad, CA, USA) in the dark for 1 h. After washing, fluorescent images of labeled cells were examined under a confocal microscope (FV300; Olympus, Tokyo, Japan).

***Assessment of mPTP Opening***

The calcein/CoCl_2_-quenching technique was applied to detect the opening of mPTP using the commercial mPTP fluorescence assay kit (GMS10095; Genmed Scientific Inc., Shanghai, China). When mPTP was closed, calcein retained within the mitochondrion shows green fluorescence along with red color of Mito Tracker, which after being merged reveals mitochondria in yellow color. In contrast, when mPTP is open, CoCl_2_ quenches the calcein which in turn is released into the cytoplasm and turns color from yellow to red. Images of cells were analyzed over 5 randomly selected fields under a confocal laser scanning microscope.

***Quantitative Real-Time Reverse Transcription (RT)-Polymerase Chain Reaction (qPCR)***

Total RNA samples were extracted from cardiac tissues and NMVCs using Trizol reagent (Invitrogen, Carlsbad, CA, USA) following the manufacturer’s instruction. Quality of the samples was determined using an Agilent 2100 Bioanalyzer (Agilent Technologies, Santa Clara, CA, USA). RNA quantity was measured by NanoDropND-8000 (Thermo Fisher Scientific, Waltham, MA, USA), and RNA integrity was assessed by standard denaturing agarose gel electrophoresis showing discrete 28s and 5s bands with minimal smears.

Complementary DNA synthesis was performed with random primers according to the manufacturer's instructions (Reverse Transcription System, Cat#1202162; Promega, USA). The SYBR Green PCR Master Mix Kit (Cat#4309155; Applied Biosystems, Foster City, CA, USA) was used for real-time PCR to quantify the target genes on the ABI 7500 fast Real Time PCR system (Applied Biosystems). GAPDH was used as an internal control in each sample. The specificity of the amplified product was monitored by its melting curve. PCR primers (**Table S3**) were designed using the Primer Premier 3.0 program (PREMIER Biosoft International, Palo Alto, CA, USA). Relative standard curves were generated by plotting the threshold value (Ct) versus the log of the total amount of cDNA that was added to the reaction. All reactions were performed in triplicate, and relative expression levels of lncR-MIAT and mRNA were calculated using the comparative cycle threshold (Ct) method (2^-ΔΔCt^).

***Western Blot Analysis***

Total protein samples were extracted from cultured cells and the peri-infarct region of left ventricular myocardium for immunoblotting analysis. Protein content was determined with the bicinchoninic acid protein assay kit ([Beyotime Institute of Biotechnology](http://www.beyotime.com/), Shanghai, China) using bovine serum albumin as the standard. Protein samples (60-80 μg) were separated in 15% SDS-PAGE gel and blotted to nitrocellulose membranes. After blocked with 5% non-fat milk, the membranes were probed with TSPO (1:500 dilution, Abcam, Cambridge, MA, USA), caspase-3 (Casp3; 1:1000 dilution, Cell Signaling Technology, Danvers, MA, USA), Bax and Bcl-2 (1:500 dilution, Proteintech, Chicago, IL, USA), β-actin (1:2000 dilution, Santa Cruz, USA), and GAPDH antibodies (1:2000 dilution, Kangcheng Inc, Shanghai, China) in 5% non-fat milk and incubated at 4°C overnight. The membrane was subsequently incubated with infrared fluorescent dye-labeled secondary antibody (Alexa Fluor; Molecular Probes, Eugene, OR, USA) for 1 h. Images of Western blot bands were obtained using Infrared Imaging System (LI-COR Biosciences, Lincoln, NE, USA) and the band density was quantified using Odyssey 3.0 software (LI-COR Bioscience, Lincoln, NE, USA) for each group and normalized to GAPDH.

***RIP immunoprecipitation***

The Magna RIP™ RNA-Binding Protein Immunoprecipitation Kit (Millipore, Darmstadt, Germany) was used to perform the RNA immunoprecipitation assay. RNA was reverse transcribed to cDNA by using the ReverTra Ace qPCR RT Kit (Toyobo, Osaka, Japan). And the real-time RT-PCR was performed to quantifiy of the target genes. The gene-specific primers sequences were as following: MIAT-FD, Forward: AGCTGCTAAGCCAGGGCCACA; Reverse: CAGCCAGGGAAAGGCAGGGAT. ***RNA-Protein Pulldown***

The Pierce™ Magnetic RNA-Protein Pull-Down Kit (Thermo, Rockford, USA) was used in RNA-Protein pulldown experiments according to manufacturer’s instructions. The proteins pulled down by MIAT-FD (function domain of lncR-MIAT), antisense of MIAT-FD (MIAT-FDas, as a negative control), or MIAT-NC (negative control) were detected by Western blot with the anti-TSPO antibody (Abcam, Cambridge, MA, USA).

***Data Analysis***

Data are expressed as mean±SEM and were analyzed with SPSS 13.0 software. Statistical comparisons among multiple groups were performed using analysis of variance (ANOVA) followed by Dunnett’s test. Student *t*-test was carried out for comparisons between two groups. A two-tailed *p*<0.05 was taken to indicate a statistically significant difference.

**
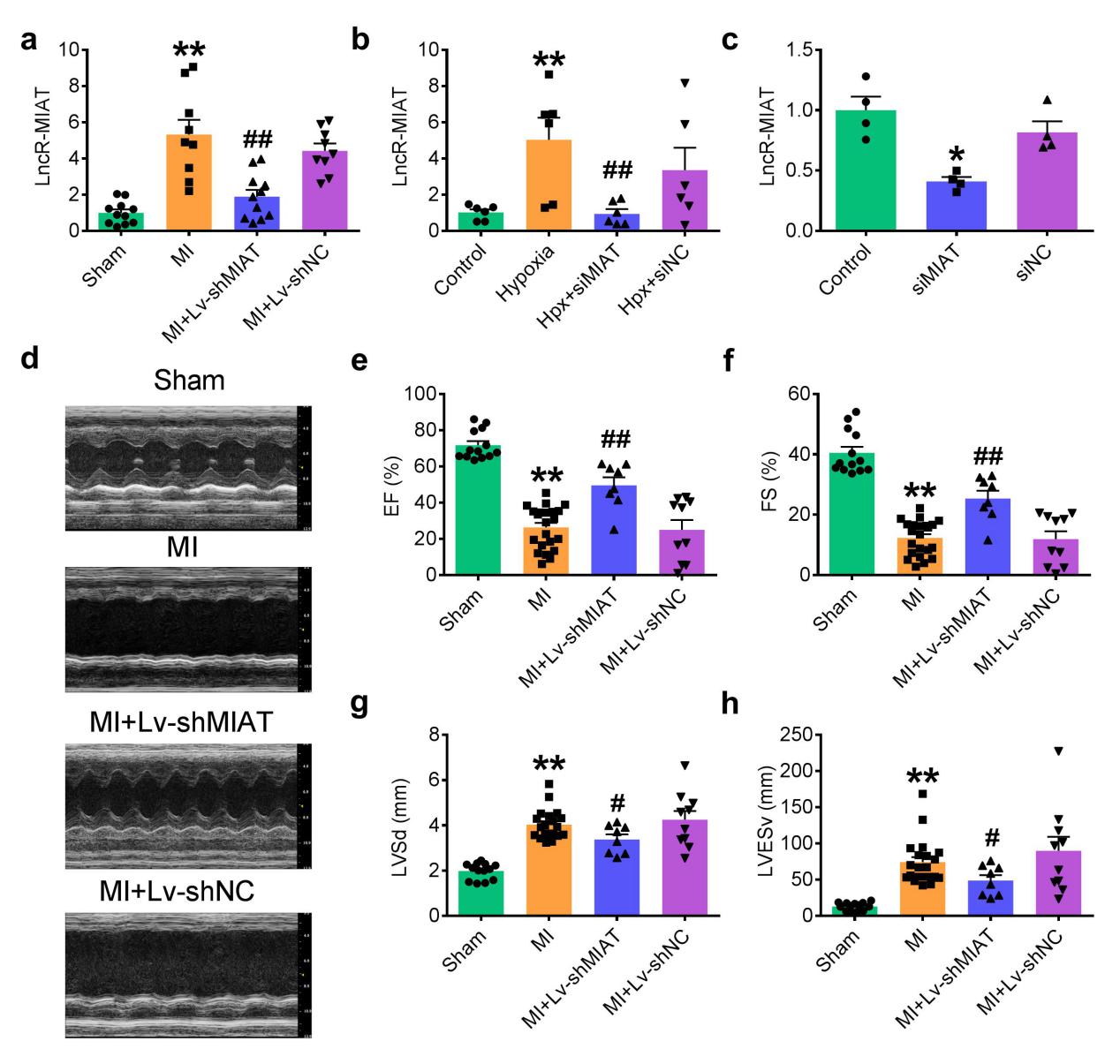
**

**Fig. S1. Long non-coding RNA Myocardial Infarction-Associated Transcript (lncR-MIAT) is upregulated in myocardial infarction (MI) and hypoxia condition.** (**a**) The expression of lncR-MIAT was significantly increased in MI hearts, which was assessed by qPCR and expressed as relative levels after normalization to their respective internal controls (same below). Note that the lentivirus carrying a shRNA for lncR-MIAT (Lv-shMIAT) the MI/Hpx-induced upregulation of lncR-MIAT, whereas the negative control shRNA (Lv-shNC) did not affect lncR-MIAT expression. ***P*<0.01 *vs.* Sham; ^##^*P*<0.01 *vs.* MI; n=9-11. (**b**) The expression of MIAT was significantly increased in neonatal mouse ventricular cell (NMVCs) cultured under hypoxia conditions (Hpx), which was essentially abolished by silencing endogenous lncR-MIAT using siRNA (siMIAT), but not by the negative control siRNA (siNC). ***P*<0.01 *vs.* Control; ^##^*P*<0.01 *vs.* Hpx; n=6. (**c**) Verifications of knockdown of endogenous lncR-MIAT by siMIAT in vitro with NMVCs, analyzed by qPCR. **P*<0.05 *vs.* Control; n=4. (**d**) Representative echocardiography images showing the cardiac dysfunction and amelioration by Lv-shMIAT in MI hearts. (**e-h**) Averaged data of cardiac contractile function determined by echocardiographic measurements on ejection fraction (EF%), fractional shortening (FS%), left ventricular systolic diameter (LVSd) and left ventricular end-systolic volume (LVESv). Note that Lv-shMIAT effectively reversed the detrimental alterations of cardiac function caused in MI mice, but Lv-shNC failed to produce any beneficial effects. ***P*<0.01 *vs.* Sham; ^#^*P*<0.05, ^##^*P*<0.01 *vs.* MI; n=8-20.


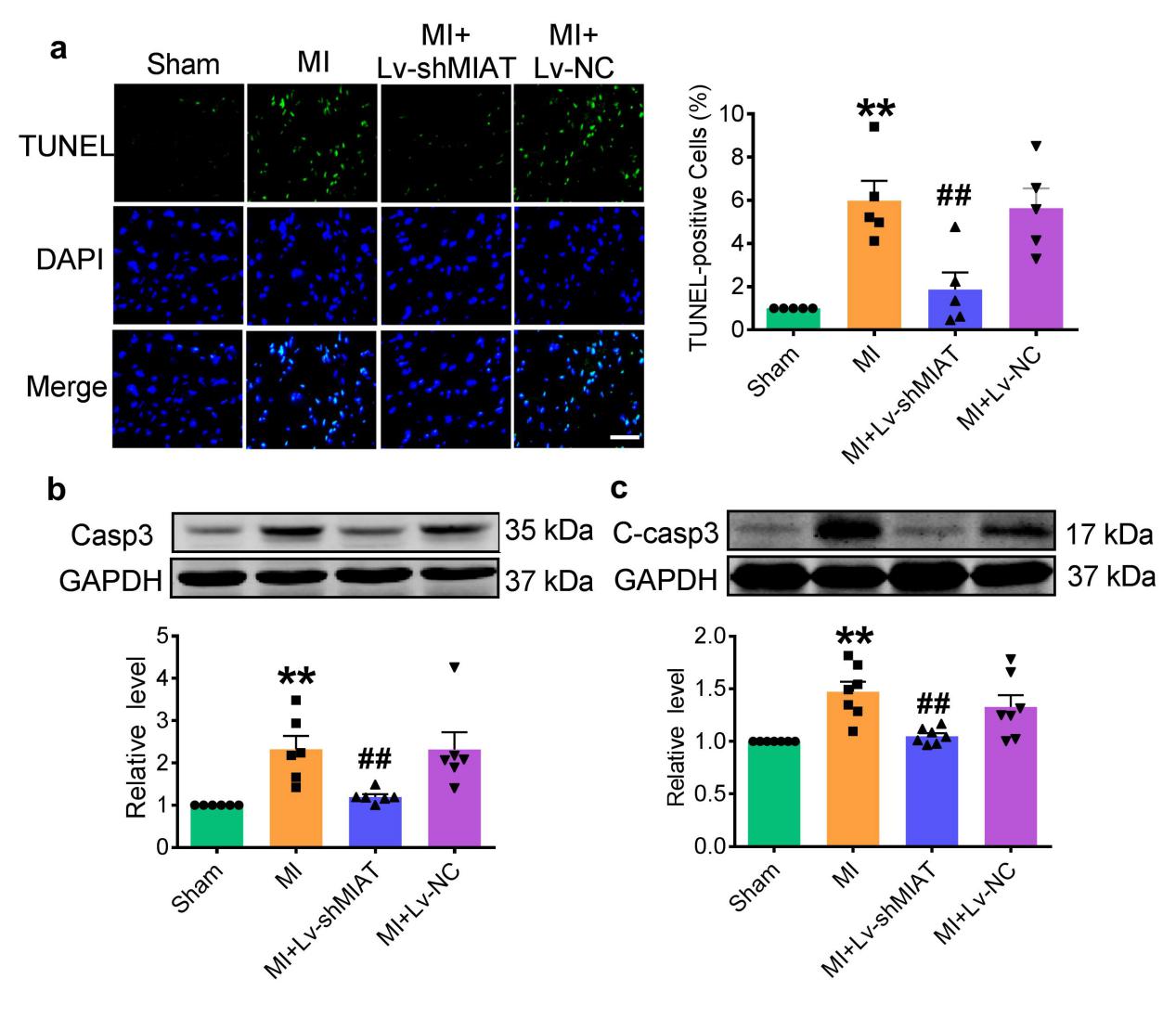


**Fig. S2. LncR-MIAT induces apoptosis in infarcted heart.** (**a**) Representative images of TUNEL staining in cardiac tissue sections from border regions showing marked increase in apoptosis in MI myocardium and rescuing of MI-induced apoptosis by Lv-shMIAT (200×magnification, left panel) and mean data of the percentage of TUNEL positive cells (right panel). The percentage of TUNEL positive cells (green) was calculated by the ratio of apoptosis cells to the total number of DAPI stained cells (blue). Lv-shNC represents the lentivirus construct carrying a scrambled control shRNA. ***P*<0.01 *vs.* Sham; ^##^*P*<0.01 *vs.* MI; n=5. (**b**) Western blot analysis showing the reversal of MI-induced upregulation of protein level of total caspase-3 (Casp3) by knocking down endogenous lncR-MIAT with Lv-shMIAT. ***P*<0.01 *vs.* Sham; ^##^*P*<0.01 *vs.* MI; n=6. (**c**) Western blot analysis showing the reversal of MI-induced upregulation of protein level of cleaved or activated caspase-3 (c-Casp3) by knocking down endogenous lncR-MIAT with Lv-shMIAT. ***P*<0.01 *vs.* Sham; ^##^*P*<0.01*vs.* MI; n=7.


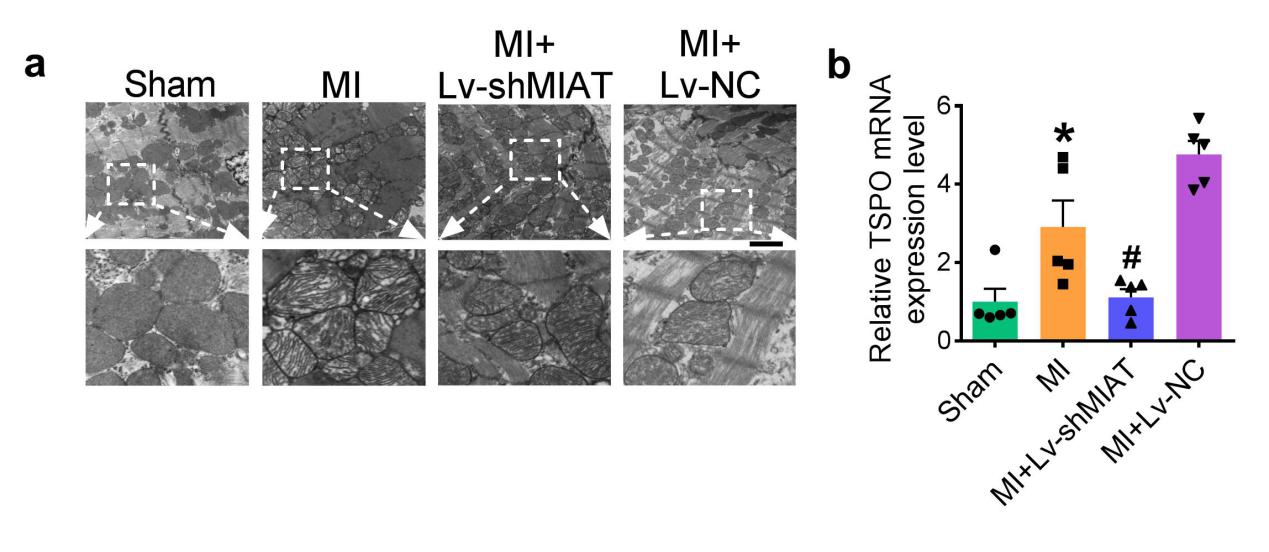


**Fig. S3. LncR-MIAT affects translocator protein (TSPO).** (**a**) Transmission electron microscope examination of mitochondrial damage (15000× magnification) in the peri-infarct zone of myocardium, as reflected by mitochondrial swelling and loss of dense, opaque and even surface of mitochondria in MI hearts that were absent in sham control hearts. Lv-shMIAT alleviated the MI-induced mitochondrial damages, but Lv-shNC did not. (**b**) Upregulation of TSPO expression at both mRNA and protein levels in MI hearts and restoration of this upregulation by lncR-MIAT knockdown, as assessed by qPCR and Western blot analysis. **P*<0.05, ****P*<0.001 *vs.* Sham; ^#^*P*<0.05, ^##^*P*<0.01 *vs.* MI; n=5.


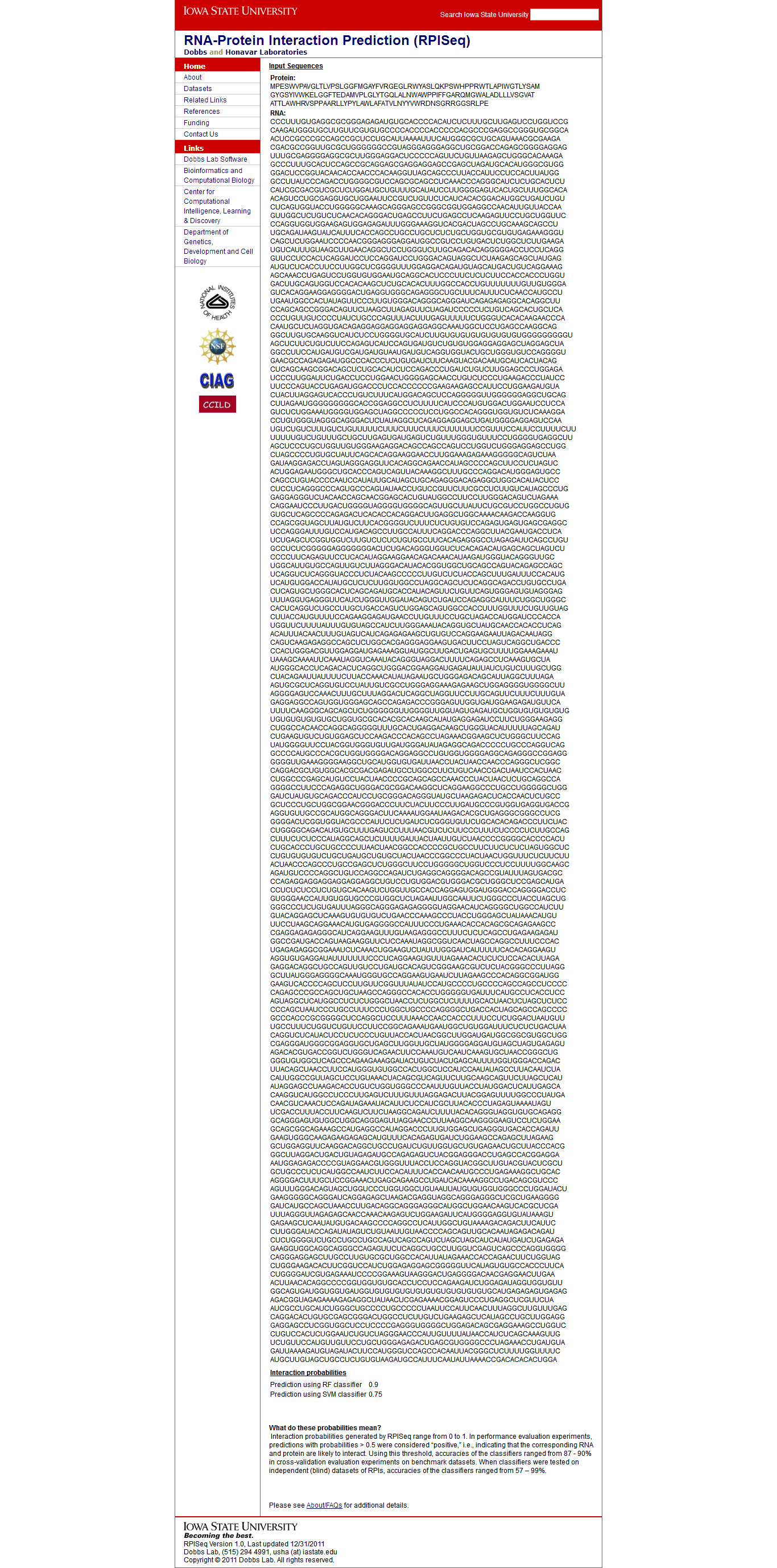


**Fig. S4. Computational analysis for lncR-MIAT:TSPO interaction.** RNA: protein binding using the RNA-Protein Interaction Prediction (RPISeq) database showing a high probability of lncR-MIAT:TSPO interaction.


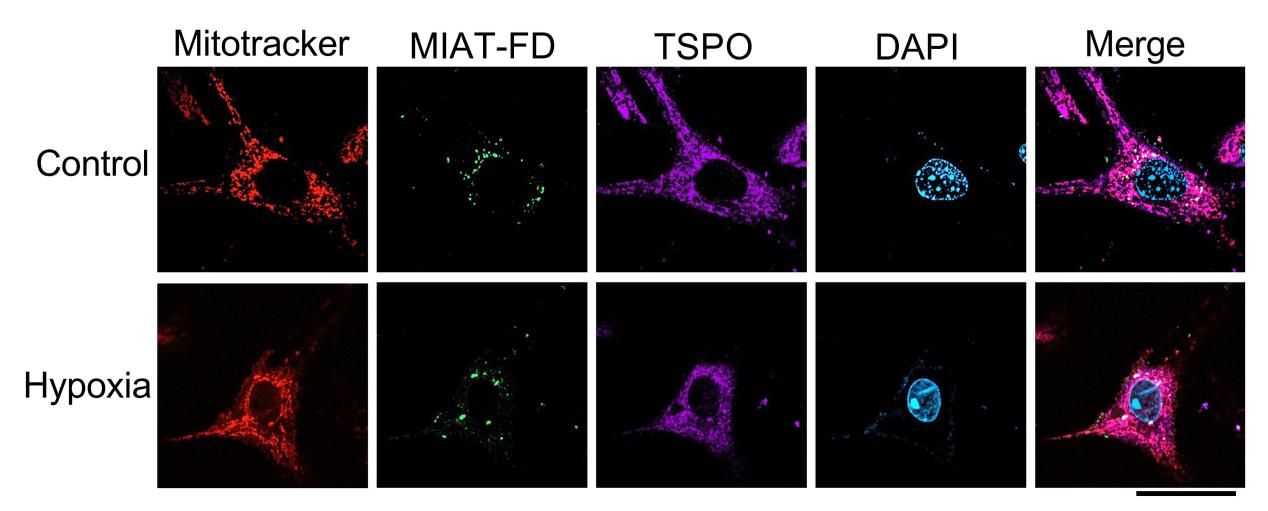


**Fig. S5.** **Co-localization of lncR-MIAT and TSPO in mitochondria**. The functional domain of lncR-MIAT (MIAT-FD in green) was primarily distributed in the rod-shaped organelles around the nucleus, and MitoRed stained the same subcellular bodies, indicating the presence of lncR-MIAT in mitochondria. TSPO (violet) was co-stained with MitoRed throughout the cytoplasm indicating its mitochondrial localization (600× magnification).


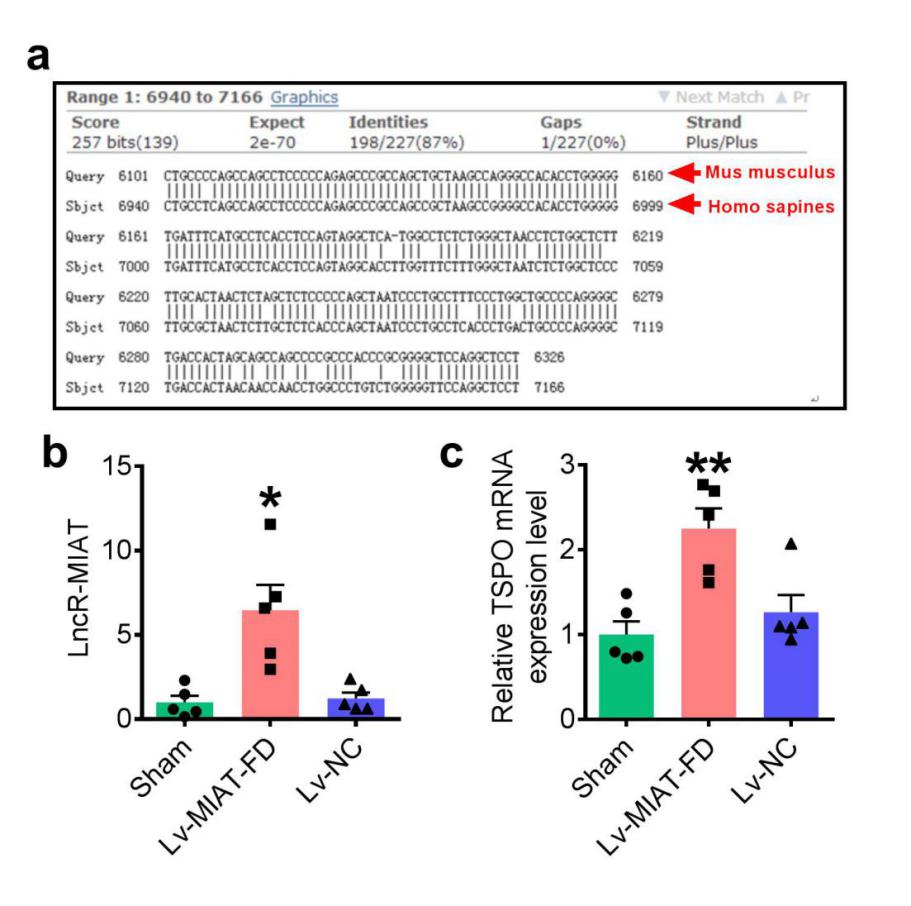


**Fig. S6. Identification and functional characterization of the functional domain of** **lncR-MIAT (MIAT-FD).** (**a**) Alignment of the homologous regions of lncR-MIAT sequences between human and mouse. (**b**) qPCR verification of infection efficiency of lentivirus carrying the functional domain of lncR-MIAT (Lv-MIAT-FD) in the myocardium of mice analyzed. Lv-NC was used as a negative control fragment for Lv-MIAT-FD. **P*<0.05 *vs.* Sham; n=5. (**c**) Overexpression of MIAT-FD increased TSPO expression at mRNA level, evaluated by qPCR. ***P*<0.01 *vs.* Sham; n=5.


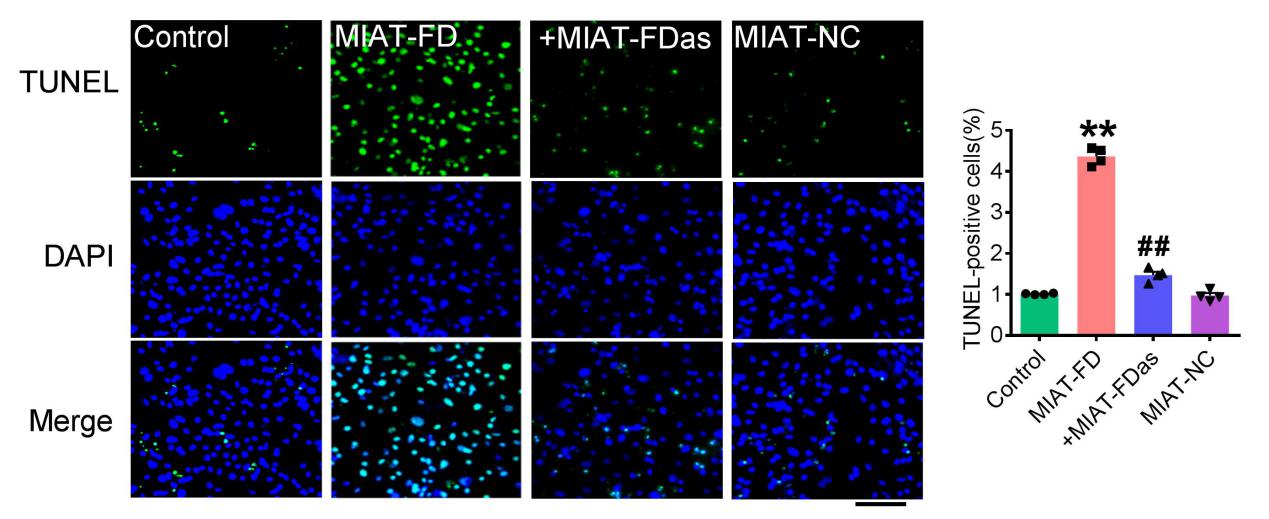


**Fig. S7.** TUNEL assay (200× magnification) demonstrating an enormous increase of apoptosis in NMCMs transfected with MIAT-FD and mitigation of the effect of MIAT-FD by its antisense oligo fragment (MIAT-FDas). Apoptosis was expressed as the percentage of TUNEL positive cells relative to DAPI stained cells. ***P*<0.01 *vs*. Control; ^##^*P*<0.01 *vs*. MIAT-FD; n=4.

**Table S1. Lv-shMIAT ameliorates cardiac dysfunction induced by myocardial infarction (MI).**

|  | Sham | MI | MI+shMIAT | MI+NC |
| --- | --- | --- | --- | --- |
| LVDd (mm) | 3.31±0.09 | 4.45±0.12^***^ | 4.60±0.19 | 4.76±0.31 |
| LVSd (mm) | 1.98±0.10 | 4.03±0.15^***^ | 3.38±0.22^#^ | 4.25±0.39 |
| LVEDv (µL) | 45.14±2.88 | 92.02±6.21^***^ | 99.35±9.35 | 111.43±17.62 |
| LVESv (µL) | 13.12±1.52 | 74.19±7.01^***^ | 48.96±7.33^#^ | 89.91±19.26 |
| EF (%) | 71.83±2.22 | 26.40±2.49^***^ | 49.60±4.44^###^ | 25.06±5.43 |
| FS (%) | 40.53±1.98 | 12.34±1.22^***^ | 25.39±2.59^###^ | 11.9±2.65 |

**Table S2. Sequences of lncR-MIAT shRNA and a negative control shRNA**

|  | Sequences |
| --- | --- |
| MIAT-sense | 5’-GCAGUUCUUAGCUCAUAUATT-3’; |
| MIAT-anti-sense | 5’-UAUAUGAGCUAAGAACUGCTT-3’; |
| NC-sense | 5’ -UUCUCCGAACGUGUCACGUTT-3’; |
| NC-anti-sence | 5’-ACGUGACACGUUCGGAGAATT-3’. |

**Table S3. Sequences of the primer pairs used for real time PCR analysis**

| Name |  | Primers |
| --- | --- | --- |
| MIAT | Forward | 5’-TGGAACAAGTCACGCTCGATT-3’ |
|  | Reverse | 5’-GGTATCCCAAGGAATGAAGTCTGT-3’ |
| β-actin | Forward | 5’ -ACTGCCGCATCCTCTTCT-3’ |
|  | Reverse | 5’-TCAACGTCACACTTCATGATGGA-3’ |
